# ﻿Three new species of *Bredia* (Sonerileae, Melastomataceae) from the Sino-Vietnamese border area

**DOI:** 10.3897/phytokeys.195.83934

**Published:** 2022-05-09

**Authors:** Jin-Hong Dai, Shi-Yue Nong, Xi-Bin Guo, Truong Van Do, Yan Liu, Ren-Chao Zhou, Ying Liu

**Affiliations:** 1 State Key Laboratory of Biocontrol and Guangdong Key Laboratory of Plant Resources, School of Life Sciences, Sun Yat-sen University, No. 135, Xin-Gang-Xi Road, Guangzhou 510275, China; 2 Guangxi Institute of Botany, Guangxi Zhuang Autonomous Region and the Chinese Academy of Sciences, Guilin 541006, China; 3 Malipo Laoshan Provincial Natural Reserve, Malipo 663600, China; 4 Vietnam National Museum of Nature, Vietnam Academy of Science and Technology, 18; 5 th; 6 Hoang Quoc Viet Road, Cau Giay, Hanoi, Vietnam; 7 Graduate University of Science and Technology, Vietnam Academy of Science and Technology, 18; 8 th; 9 Hoang Quoc Viet Road, Cau Giay, Hanoi, Vietnam

**Keywords:** *Bredia*, karst, Melastomataceae, phylogeny, taxonomy

## Abstract

*Brediabullata*, *B.enchengensis*, and *B.nitida* (Sonerileae, Melastomataceae), three species occurring in Sino-Vietnamese limestone karst regions, are described as new. Molecular phylogenetic analyses and morphological divergence indicate that these species are well separated from their close relatives in *Bredia*, justifying their recognition as distinct species. *Brediabullata* is unique in its interveinal areas prominently bullate each with an apical seta, a character otherwise never recorded in the genus. *Bredianitida* resembles *B.malipoensis* in habit, leaf shape, and inflorescence morphology, but differs in the glabrescent and nitid adaxial leaf surface (vs. densely pubescent and subvelvety), ovate-elliptic or elliptic calyx lobes (vs. triangular to semiorbicular), and white petals (vs. purplish-red). *Brediaenchengensis* is closest to *B.longiradiosa*, but easily recognized by its prostrate habit (vs. erect), the yellowish-green, membranous and fragile leaves (vs. green or dark green, papery), and white anthers (vs. pink to purplish). These new discoveries show that further botanical exploration is warranted in the remote Sino-Vietnamese bordering region.

## ﻿Introduction

Karst is a kind of landscape characterized by a variety of closed surface depressions, a well-developed underground drainage system and a paucity of surface streams (Ford and Williams 2007). The complex terrains and variable climatic conditions on karsts provide numerous ecological niches that harbor a rich biodiversity (Clements et al. 2006). The vast karst terrain stretching across southern China and northern Vietnam connects two global biodiversity hotspots, viz. south-central China and Indo-Burma. It harbors remarkable biodiversity and a high level of endemism (Zhu 2007) and has been considered the model for karst studies (Sweeting 1978). As karst environments in these areas are often remote and under significant threats due to human activity, biodiversity survey and conservation are extremely urgent.

**Figure 1.**
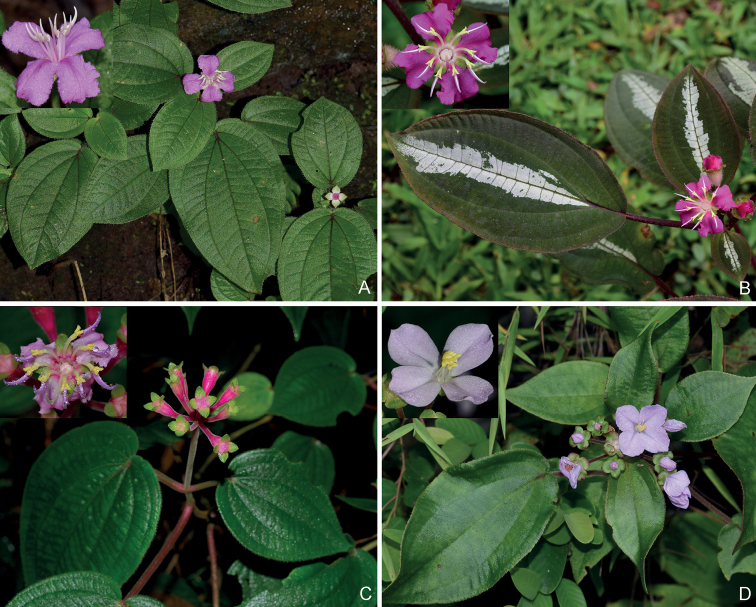
Species of *Bredia* adapted to limestone karst habitats **A***B.latisepala*, Ying Liu 557 (SYS) **B**B.longiradiosavar.longiradiosa, Ying Liu 486 (SYS) **C***B.malipoensis*, Yunnan Expedition Team 1073 (PE), photographs by Bing Liu (PE) **D***B.reniformis*, Ying Liu 748 (SYS).

**Figure 2.**
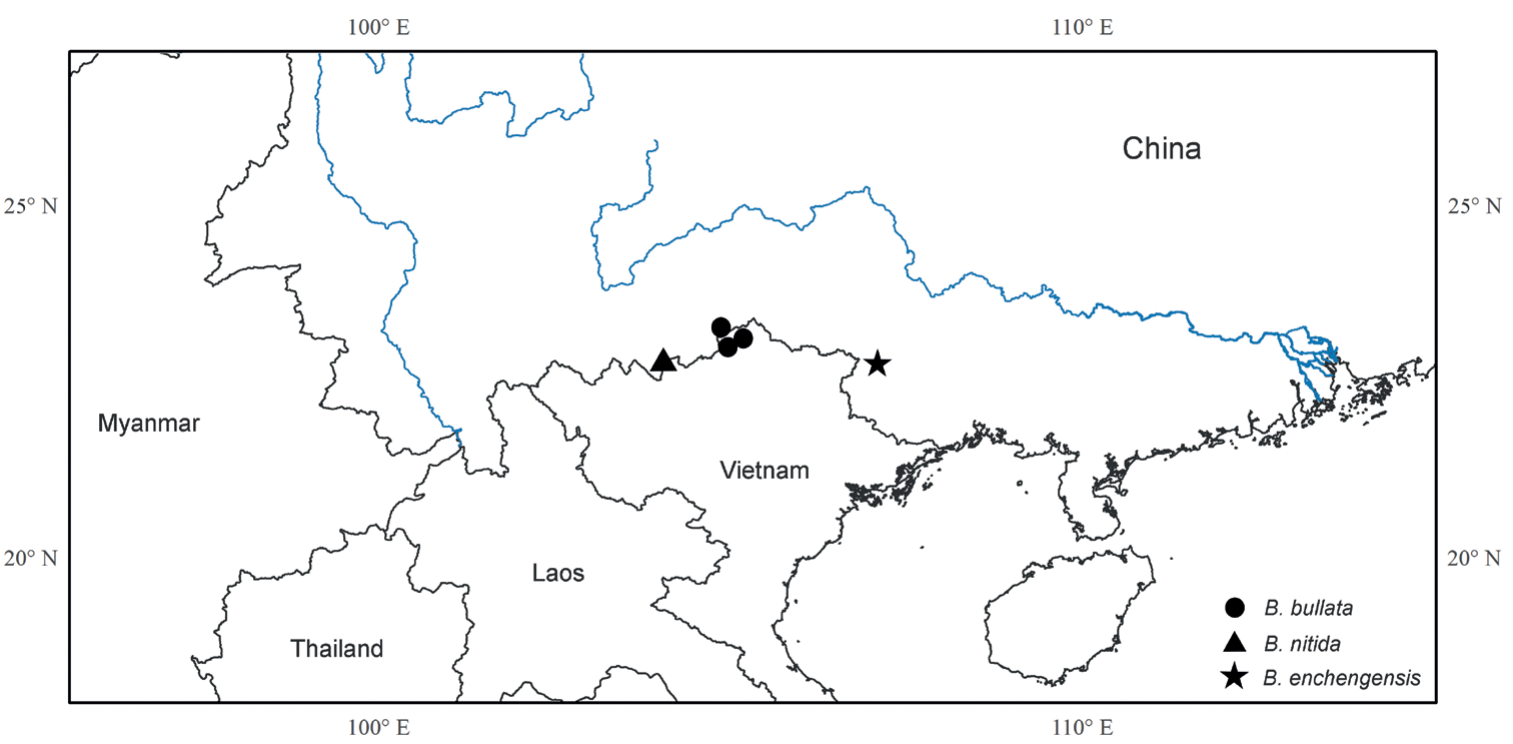
Distribution of *Brediabullata* (solid circle), *B.nitida* (triangle), and *B.enchengensis* (star).

**Figure 3.**
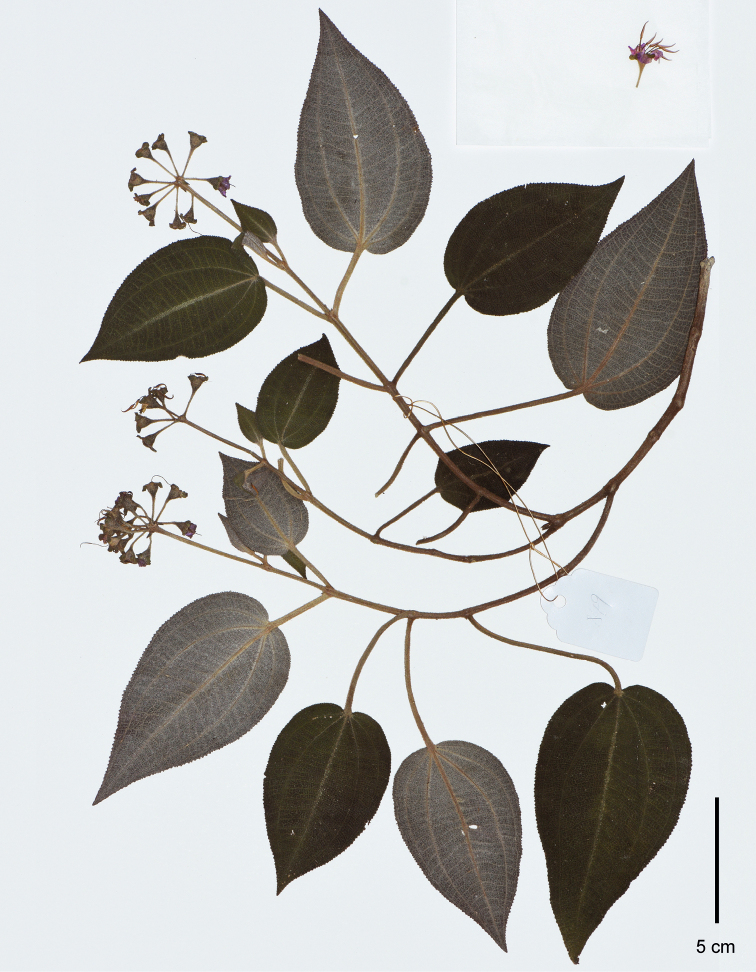
Holotype of *Brediabullata*, Jin-hong Dai and Ying Liu 849 (PE). Scale bar: 5 cm.

**Figure 4.**
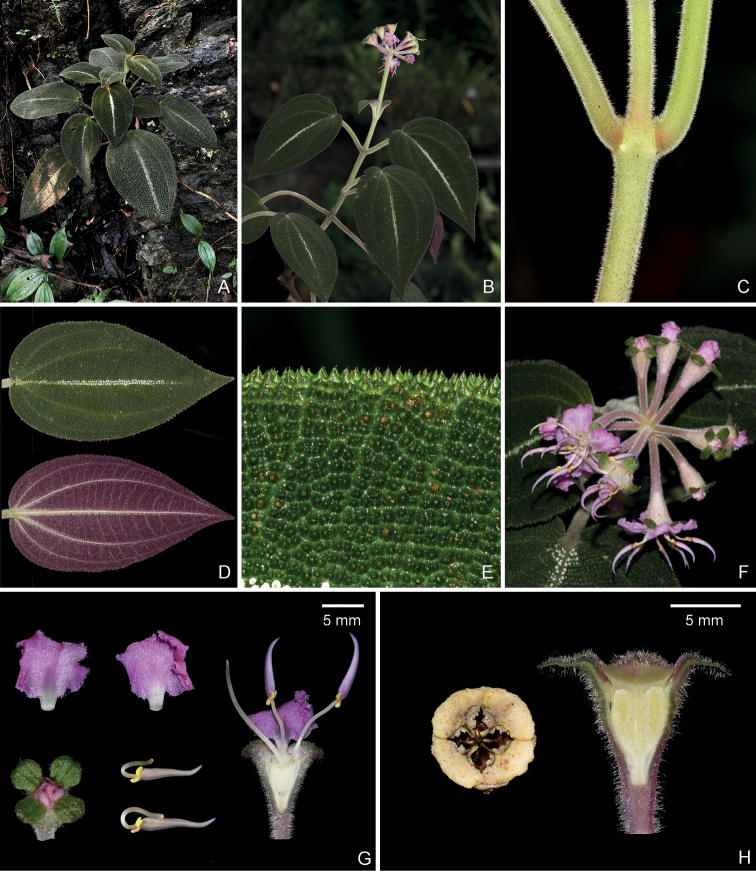
*Brediabullata***A** habit **B** a flowering branch **C** a branchlet showing spreading hairs with and without glandular head **D** adaxial (top) and abaxial (bottom) leaf surfaces **E** closeup of adaxial leaf surface showing interveinal areas prominently bullate, each bulla with an apical seta **F** flowering inflorescence **G** two petals (upper left and middle), bud showing rounded calyx lobes (lower left), inner and outer stamens (lower middle), and longitudinal section of flower (right) showing isomorphic stamens and ovary crown **H** top view of old capsule (left) and longitudinal section of young fruit showing enlarged ovary crown (right). Scale bars: 5 mm (**G, H**). All from Jin-hong Dai and Ying Liu 849 (A, PE, SYS).

**Figure 5.**
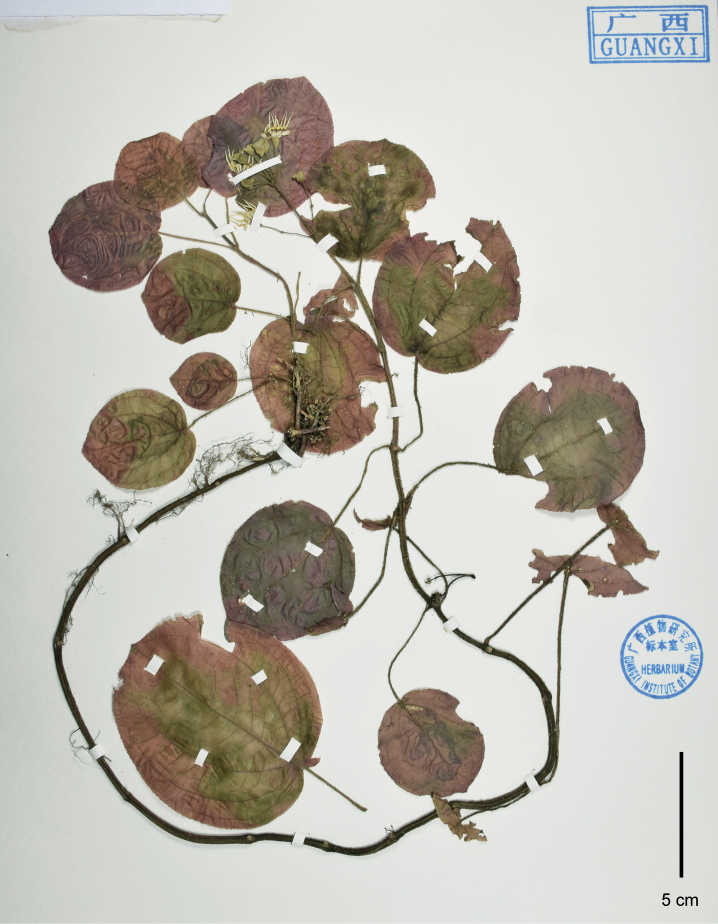
Holotype of *Brediaenchengensis*, Shi-yue Nong and Jin-hong Dai EC20210708001 (IBK). Scale bar: 5 cm.

**Figure 6.**
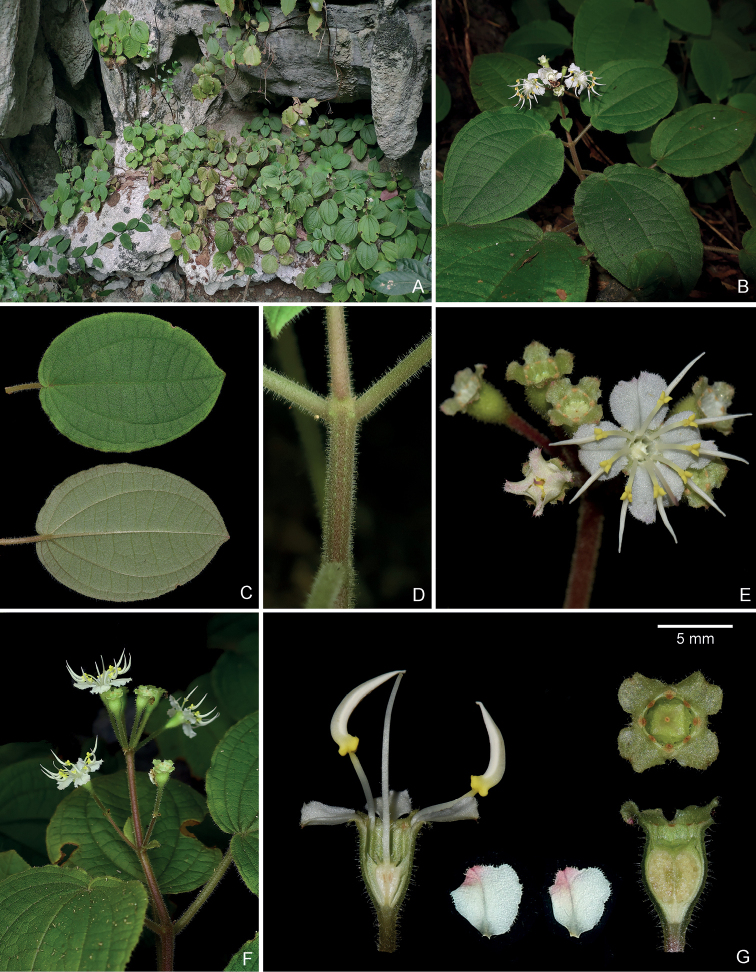
*Brediaenchengensis***A** habitat and habit **B** flowering branch **C** adaxial (top) and abaxial (bottom) leaf surfaces **D** branchlet showing spreading glandular hairs **E** terminal cyme **F** cymose panicle **G** longitudinal section of flower (left) showing isomorphic stamens, two petals (lower middle), and top view (upper right) and longitudinal section (lower right) of young fruit showing broadly ovate to reniform calyx lobes and ovary crown. Scale bar: 5 mm (**G**). All from Shi-yue Nong and Jin-hong Dai EC20210708001 (A, IBK, PE, SYS).

**Figure 7.**
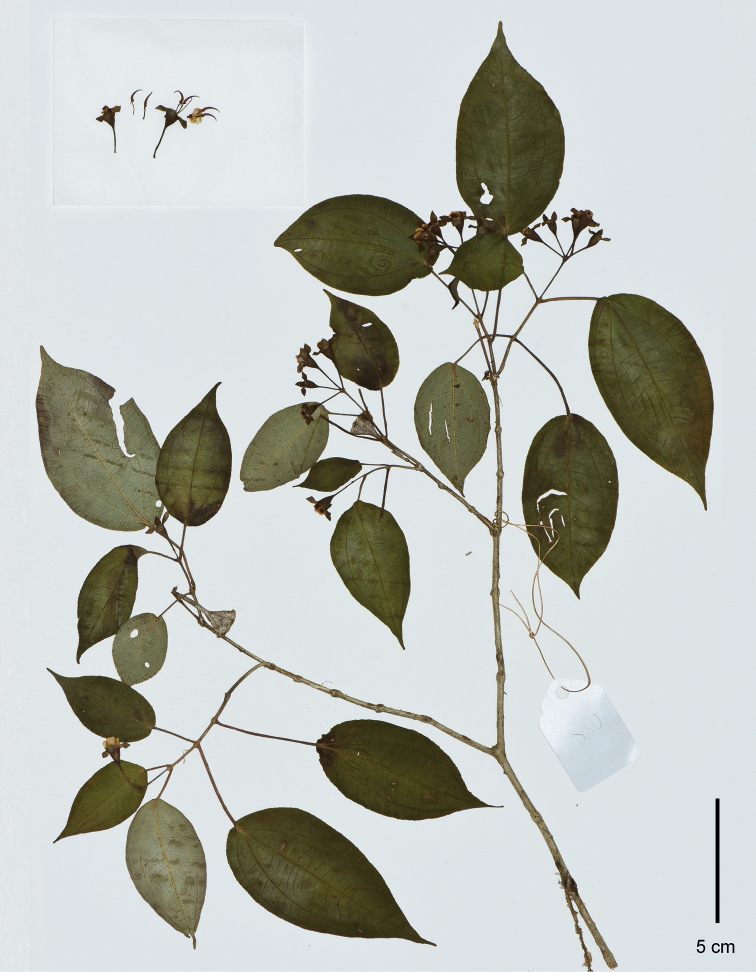
Holotype of *Bredianitida*, Jin-hong Dai and Ying Liu 850 (PE). Scale bar: 5 cm.

**Figure 8.**
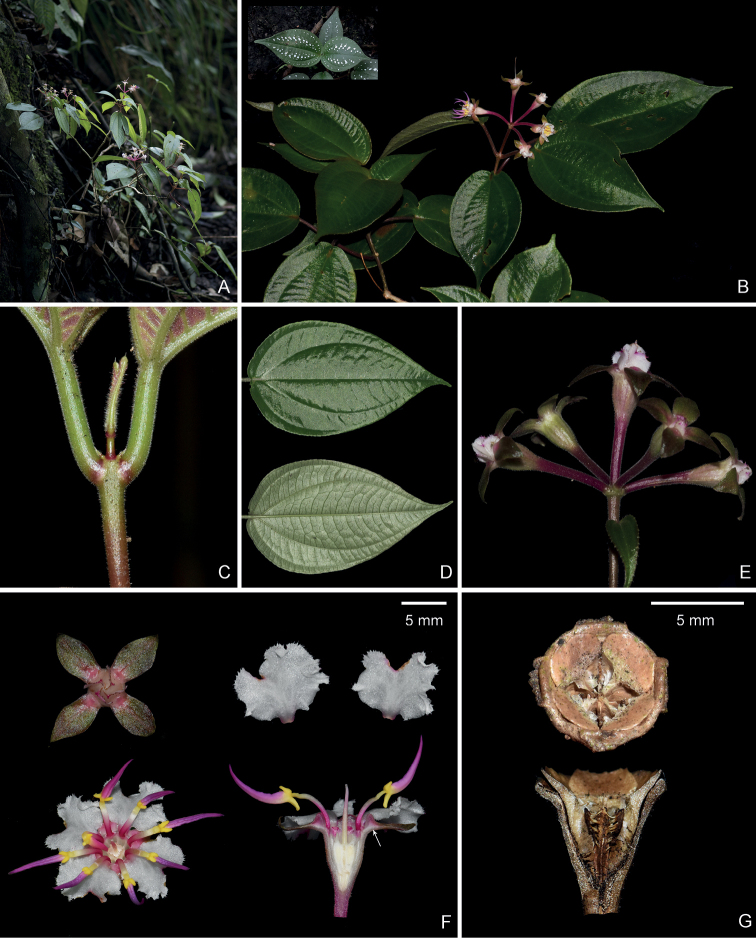
*Bredianitida***A** habit **B** young leaves with white patches (insert) and flowering branch **C** sparsely puberulous young branchlet with spreading minute hairs **D** adaxial (top) and abaxial (bottom) leaf surfaces **E** flowering inflorescence **F** top view of flower bud showing ovate-elliptic calyx lobes (upper left), two petals (upper right), top view of flower (lower left), and longitudinal section of flower (lower right) showing the isomorphic stamens and thick basal protuberance (indicated by arrow) on calyx lobe **G** top view (top) and longitudinal section (bottom) of old capsule showing enlarged ovary crown. Scale bars: 5 mm (**F**, **G**). All from Jin-hong Dai and Ying Liu 850 (A, PE, SYS).

*Bredia* Blume (Melastomataceae) as currently circumscribed contains 24 species distributed from central and southern mainland China, Taiwan, northern Vietnam, to the Ryukyu Islands and Yakushima, Japan (Zhou et al. 2019a; Wen et al. 2019; Dai et al. 2020; He et al. 2020). Five species of *Bredia*, namely *B.latisepala* (C. Chen) R. Zhou & Ying Liu, *B.longearistata* (C. Chen) R. Zhou & Ying Liu, *B.longiradiosa* C. Chen ex Govaerts, *B.malipoensis* D. H. Peng, S. J. Zeng & Z. Y. Wen, and *B.reniformis* C. M. He, Y. H. Tong & S. J. Zeng, are restricted to limestone karst areas. These species share obvious resemblance in their isomorphic stamens and undulate petal margin ciliate with glandular hairs and thus are easily distinguished from the remaining species of the genus (Fig. 1). The only exception is *B.reniformis*, which does not have an undulate petal margin (Fig. 1D). Close relationships among the karst species were consistently recovered in previous phylogenetic studies based on nuclear ribosomal internal transcribed spacer (nrITS) and plastome sequences (Zhou et al. 2019a, b, c; Dai et al. 2020). From 2019 to 2021, multiple field expeditions were made to karst areas in southern Guangxi, southeastern Yunnan, and northern Vietnam. In the process, we encountered three species of *Bredia* with isomorphic stamens and undulate and ciliate petal margin that were morphologically distinct from limestone species. As shown in Fig. 2, the new taxa were found in three localities from Malipo County, Yunnan Province, China and Quan Ba District, Ha Giang Province, Vietnam (*B.bullata* J. H. Dai & Ying Liu; Figs 3, 4); in one locality from Daxin County, Guangxi Province, China (*B.enchengensis* J. H. Dai, Yan Liu & S. Y. Nong; Figs 5, 6); and in one locality from Hekou County, Yunnan Province, China (*B.nitida* J. H. Dai & Ying Liu; Figs 7, 8).

In this study, we inferred the phylogenetic position of the plants in question and then compared them with their close relatives in *Bredia* to evaluate their specific status. To this end, phylogenetic analyses were performed using sequence data of three nuclear markers (nrITS, *Dbr1*, and *SOS4a*) and one chloroplast intergenic spacer (*trnV*–*trnM*), sampling all species recorded in *Bredia*. The results confirmed our suspicion that these plants represented species of *Bredia* new to science. A key is provided for the karst species.

## ﻿Materials and methods

Morphological data for the new species and previous recorded karst species were obtained through field expeditions, herbarium specimens (A, E, GXMI, IBK, IBSC, PE, SYS, VNMN) and literature (Chen 1984; Chen and Renner 2007; Wen et al. 2019; He et al. 2020) surveys as well as by observing living individuals in the facilities of Sun Yat-sen University.

To infer the phylogenetic position of *B.bullata*, *B.nitida*, and *B.enchengensis*, the type species of related genera (*Blastus* Lour., *Fordiophyton* Stapf, *Phyllagathis* Blume, *Tashiroea* Matsum. ex Ito & Matsum.), and all 24 species so far recorded in *Bredia* were included in the analyses. *Tashiroeayaeyamensis* Matsum. was selected as the outgroup according to Zhou et al. (2019a, b, c). The source of the materials and GenBank accession numbers are given in Suppl. material 1.

Total DNA was extracted from fresh leaves using the modified CTAB procedure (Doyle and Doyle 1987). For amplification and sequencing of *SOS4a*, we used two primers (*SOS4a*-F: 5´-CAAGAAGGTGAGATCATCCAAA-3´, *SOS4a*-R: 5´-TAGTTTTGGCCTGCAATGCT-3´) adapted from Reginato and Michelangeli (2016). Primers published in Zhou et al. (2020) were used for *Dbr1* and universal primers for nrITS and *trnV*–*trnM* (White et al. 1990; Hwang et al. 2000).

Sequences of four genes were aligned using MAFFT v.7.307 (Katoh and Standley 2013) and concatenated. Maximum likelihood (ML) analysis was performed in IQ-TREE v.2.1.4 (Nguyen et al. 2015). The optimal partitioning scheme and best-fitting model for each partition (Suppl. material 2) were selected using ModelFinder (Kalyaanamoorthy et al. 2017) under the Bayesian Information Criterion (BIC). Node support was evaluated by 1000 replicates of ultrafast bootstrap (UFBS) (Minh et al. 2013) and SH-aLRT test. For Bayesian inference (BI) analysis, we used PartitionFinder v.2.1.1 (Lanfear et al. 2017) for partitioning and model selection (Suppl. material 2). BI analysis was conducted in MrBayes v.3.2.6 (Huelsenbeck and Ronquist 2001). Two independent Markov chain Monte Carlo analyses (MCMC) were performed with four simultaneous chains of 2,000,000 generations sampling one tree every 100 generations. We verified that the average deviation of split frequencies had reached a value below 0.01 at the end of MCMC analyses. The first 25% of trees were discarded as burn-in and the remaining were used to construct a majority-rule consensus tree with Bayesian posterior probabilities (PP). Effective sample sizes (ESS) for all parameters and statistics were assessed using Tracer v.1.7.1 (Rambaut et al. 2018). Maximum parsimony (MP) analysis was carried out in PAUP v.4a165 (Swofford 2003). A heuristic search strategy was conducted of 1000 random addition replicates, with the tree-bisection-reconnection (TBR) branch swapping algorithm and MultTrees on. Maxtree was set to 500. We evaluated node support (BSMP) by 1000 bootstrap replicates of 1000 random additions.

## ﻿Results

**Figure 9.**
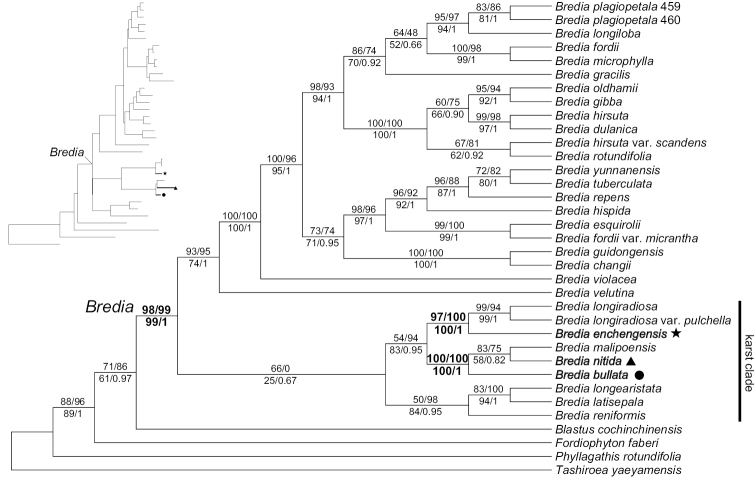
Phylogenetic position of *Brediabullata*, *B.nitida*, and *B.enchengensis*. Maximum likelihood (ML) phylogenetic tree based on combined dataset of nrITS, *Dbr1*, *SOS4a*, and *trnV*–*trnM* sequences. Numbers above branches are ultrafast bootstrap (left) and SH-aLRT test (right) obtained from ML analy-sis, and those below branches are Bayesian posterior probabilities (right) and bootstrap values (left) resulting from maximum parsimony analyses. The new species are noted in bold.

The aligned sequence matrix contained 2536 characters. The optimal partitioning scheme contained three partitions, the statistics of which were summarized in Suppl. material 2. Trees from BI, ML, and MP analyses had identical topologies. The tree resulting from ML analysis is shown in Fig. 9, with PP, BSMP, UFBS, and support values from SH-aLRT test indicated at nodes. *Brediabullata*, *B.nitida*, and *B.enchengensis* formed a strongly supported clade with the other 24 species of *Bredia* (PP = 1; BSMP = 99%; SH-aLRT test = 99%, UFBS = 98%). Within *Bredia*, the three new taxa formed a clade with the other karst species (karst clade, Fig. 9), although with low support (PP = 0.67; BSMP = 25%; SH-aLRT test = 0%, UFBS = 66%). *Brediaenchengensis* was recovered as sister to *B.longiradiosa* (PP = 1; BSMP = 100%; SH-aLRT test = 100%, UFBS = 97%), while *B.nitida* and *B.bullata* constituted a well resolved clade with *B.malipoensis* (PP = 1; BSMP = 100%; SH-aLRT test = 100%, UFBS = 100%).

## ﻿Discussion

Phylogenetic data and morphology confirmed that *B.bullata*, *B.nitida*, and *B.enchengensis* belong in *Bredia*. All three species have cordate leaf blades, cymose inflorescences, isomorphic stamens, gibbous anthers and enlarged ovary crowns, all of which are typical of *Bredia* (Figs 3–8). In the present phylogenetic analyses (Fig. 9), the limestone species of *Bredia* formed a clade containing four subclades, viz. *B.reniformis*, *B.latisepala*-*B.longearistata*, *B.longiradiosa*-*B.enchengensis*, and *B.malipoensis*-*B.nitida*-*B.bullata*. Nevertheless, the karst clade is still weakly supported, as well as the relationships among its four subclades. Further molecular sampling is desired to improve these phylogenetic relationships.

Among the three species in question, *B.enchengensis* was well resolved as sister to *B.longiradiosa* (Fig. 9). It resembles *B.longiradiosa* in the somewhat broadly ovate leaf blade, inflorescence often an umbellate cyme, undulate petals with ciliate margin, and isomorphic stamens, but differs markedly from the latter in the prostrate habit (vs. erect), densely pubescent stem (vs. sparsely villous or glabrescent), yellowish-green, membranous and fragile leaves (vs. green or dark green, papery), and white anthers (vs. pink or purplish) (Figs 1B, 6). The remaining two species, namely *B.bullata* and *B.nitida*, formed another karst subclade in the genus with *B.malipoensis* (Fig. 9). *Brediabullata* is distinct in its strongly sunken adaxial leaf veins with interveinal areas prominently bullate each with a short apical seta (Fig. 4E), a character otherwise never recorded in the genus. *Bredianitida* shares general similarities with *B.malipoensis* in leaf shape and morphology of the inflorescence, petals, and stamens, but is easily distinguished from the latter in the often glabrescent stem and leaves at maturity (vs. densely pubescent), nitid upper leaf surface (vs. subvelvety), ovate-elliptic or elliptic calyx lobes (vs. triangular to semiorbicular), and white petals (vs. purplish-red) (Figs 1C, 8). Based on the phylogenetic data and morphological divergence, *B.bullata*, *B.nitida*, and *B.enchengensis* should be recognized as distinct species in *Bredia*.

The Sino-Vietnamese limestone karst region provides a multitude of habitats, such as cliffs, caves, and shaded fissures/crevices (Schindler 1982; Xu 1995; Zhu 2007). For some calciphilous herbaceous plant groups with low vagility, such isolated habitats/microhabitats likely promote allopatric speciation and a steady accumulation of species over time, resulting in a high diversity of narrowly endemic species (Hughes and Hollingsworth 2008; Chung et al. 2014). *Aspidistra* Ker Gawl. (e.g., Liu et al. 2011), *Begonia* L. (e.g., Chung et al. 2014), *Impatiens* L. (e.g., Xue et al. 2020), and *Primulina* Hance (e.g., Kong et al. 2017) are among the most famous examples. The Sino-Vietnamese limestone areas, where seven species of *Bredia* have been recorded, is a diversification center for the genus. These species are capsule-fruited and disperse their seeds by raindrops and wind, often within a short distance from the mother plant. Current data indicate that geographic isolation is likely the primary mode of species diversification, in a group with limited distribution range or even site-endemics. The Sino-Vietnamese karst areas are hotspots of species richness and endemism and have been an important source of vascular plant novelties in the past 20 years (Du et al. 2020; Qian et al. 2020). The remote border regions should be further explored to fully unravel the rich biodiversity there.

## ﻿Taxonomic treatment

### ﻿ Bredia bullata

Taxon classificationPlantaeMyrtalesMelastomataceae

J. H. Dai & Ying Liu sp. nov.

1F95ABCF-FEF1-5C6F-8730-CB39B4DA7255

urn:lsid:ipni.org:names:77297481-1

Figs 3 , 4

#### Type.

China. Yunnan Province: Malipo County, Ba-bu Town, Da-nong Village, 1,300 m, under forests, on limestone rocks, 30 May 2020, Jin-hong Dai and Ying Liu 849 (holotype: PE; isotypes: A, SYS).

#### Diagnosis.

Distinguished in *Bredia* by its adaxially strongly sunken leaf veins (vs. veins not sunken), with interveinal areas prominently bullate each with an apical seta (vs. smooth, not bullate).

#### Description.

Shrubs, 0.4–1.0 m tall. Stems erect and branched, terete, densely pubescent with 0.5–1 mm long, spreading, uniseriate to multiseriate hairs with or without a glandular head. Leaves opposite; petiole 3–12.5 cm long, puberulous with 0.5 mm long, spreading and often uniseriate hairs with or without a glandular head; blade ovate-cordate to elliptic-ovate, 4–22 × 2–12.5 cm, papery, secondary veins 2 or 3 on each side of midvein, all veins strongly sunken adaxially and prominent abaxially, with interveinal areas prominently bullate, each with an apical seta, adaxial surface green to dark green, sometimes with white zones along the midvein, sparsely puberulous with minute appressed uniseriate hairs, abaxial surface pale green to purplish, densely villous with uniseriate hairs, base cordate, margin ciliate and densely serrulate with each tooth having a terminal seta, apex acute or short acuminate. Inflorescence terminal, a cyme or cymose panicle, 8–27-flowered, peduncle 3.5–6.5 cm long, densely puberulous. Flowers bisexual, radial but androecium slightly bilateral, 4-merous, pedicles, hypanthium and calyx lobes densely puberulous; pedicels 0.6–1.7 cm long; hypanthium yellowish-green to purplish, funnel-shaped, 4–7 × 4–6 mm; calyx lobes 4, orbicular, 3 × 3 mm; petals 4, pink, broadly obovate to rounded, ca. 1.0 cm long, margin undulate and ciliate with glandular hairs, apex oblique; stamens 8 in two whorls, isomorphic, subequal in length with the outer whorl slightly longer than the inner one, filaments ca. 6–9 mm long, bent with the anthers to one side of the flower, anthers lanceolate, 6–8 mm long, purplish-pink, connective forming a 1 mm long, yellow dorsal spur and 2 yellow ventral lobes; ovary half inferior, locules 4, apex of ovary with membranous crown, crown margin ciliate with glandular hairs; style ca. 1.2 cm long, basally sparsely puberulous. Capsule 7 × 5 mm, funnel-shaped; placentation axial, placentas non-thready; seeds numerous, ca. 1 mm long, cuneate.

#### Phenology.

Flowering May to June, fruiting June to August.

#### Etymology.

The specific epithet is based on the bullate leaves.

#### Distribution.

*Brediabullata* is currently known from Malipo County, Yunnan Province, China and Quan Ba District, Ha Giang Province, northern Vietnam (Fig. 2), occurring in forests on limestone slopes near mountain tops and on cliffs of moist limestone caves at 1,000–1,400 m.

#### Additional specimens examined.

Vietnam. Ha Giang Province: Quan Ba District, Bat Dai Son Commune, Pai Chu Phin Village, Bat Dai Son Nature Reserve, 23.137864N, 104.999178E, 1,300 m, 5 June 2021, Do Van Truong DVT420 (VNMN); Tung Vai Commune, Kho My Village, Kho My limestone cave, 23.092797N, 104.905840E, 1,164 m, 6 June 2021, Do Van Truong DVT464 (VNMN).

### ﻿ Bredia enchengensis

Taxon classificationPlantaeMyrtalesMelastomataceae

J. H. Dai, Yan Liu & S. Y. Nong sp. nov.

7DC826B6-D3F1-53D9-AA71-B6574FBA21AF

urn:lsid:ipni.org:names:77297482-1

Figs 5 , 6

#### Type.

China. Guangxi Province: Daxin County, En-cheng Town, near Shang-ren Village, 234 m, on steep cliff of a limestone hill, 8 July 2021, Shi-yue Nong and Jin-hong Dai EC20210708001 (holotype: IBK; isotypes: A, PE, SYS).

#### Diagnosis.

Resembles *B.longiradiosa* in leaf shape and morphology of the inflorescence, petals and stamens but differs in its prostrate habit (vs. erect), densely pubescent stem (vs. sparsely villous or glabrescent), yellowish-green, membranous and fragile leaves (vs. green or dark green, papery), and white anthers (vs. pink to purplish).

#### Description.

Herbs, 8–20 cm tall. Stems to 80 cm long, branched, terete, densely pubescent with minute uniseriate hairs and 1 mm long, spreading, multiseriate glandular hairs, prostrate with adventitious roots at middle and lower parts, with the distal part (1 to 3 internodes) erect or ascending. Leaves opposite, equal to unequal; petiole 2.1–12.7 cm long, pubescent as the stem; blade broadly ovate-cordate to cordate-orbicular, 3–17 × 2.7–14 cm, membranous and fragile, pubescent on both surfaces, adaxial surface yellowish-green, abaxial surface pale green or reddish, secondary veins 3 or 4 on each side of midvein, base cordate, margin subentire, ciliate, apex acute. Inflorescence a terminal cyme, rarely cymose panicle, (1)3–13-flowered, peduncle 1.5–5.9 cm long, pubescent. Flowers bisexual, radial but androecium slightly bilateral, 4-merous, pedicles, hypanthium and calyx lobes pubescent; pedicels 0.6–2 cm; hypanthium light green, funnel-shaped, 4–6 × 3–4 mm; calyx lobes 4, broadly ovate to reniform, 2–3.5 × 3–5 mm, margin undulate; petals 4, white, sometimes pinkish at the apex, suborbicular, 2.5–7 mm long, margin undulate and ciliate with glandular hairs, apex oblique; stamens 8 in two whorls, isomorphic, equal in length, filaments 5–6 mm long, anthers lanceolate, 6–8 mm long, white, connective forming a yellow dorsal tubercle and 2 yellow ventral lobes; ovary half inferior, locules 4, apex of ovary with membranous crown, crown margin ciliate with glandular hairs; style 1.1–1.8 cm long, basally sparsely puberulous. Capsule 7 × 5 mm, funnel-shaped; placentation axial, placental column distally unhorned, placentas non-thready; seeds numerous, ca. 0.8 mm long, cuneate.

#### Phenology.

Flowering June to July, fruiting July to August.

#### Etymology.

The specific epithet is based on the name of the town, En-cheng, where *B.enchengensis* is discovered.

#### Distribution.

*Brediaenchengensis* is currently known only from Daxin County, Guangxi Province, China (Fig. 2). It occurs in forests on steep, arid limestone cliffs at 234 m.

### ﻿ Bredia nitida

Taxon classificationPlantaeMyrtalesMelastomataceae

J. H. Dai & Ying Liu sp. nov.

3246087C-E4F6-5698-AD41-1E928C29E2EE

urn:lsid:ipni.org:names:77297483-1

Figs 7 , 8

#### Type.

China. Yunnan Province: Hekou County, Nan-xi Town, Qin-cai-tang Village, 849 m, under forests, on limestone slope, 31 May 2020, Jin-hong Dai and Ying Liu 850 (holotype: PE; isotypes: A, SYS).

#### Diagnosis.

Resembles *B.malipoensis* in leaf shape and morphology of the inflorescence, petal margin, and stamens but differs in the stem and leaves often glabrescent when mature (vs. densely pubescent), nitid upper leaf surface (vs. subvelvety), ovate-elliptic or elliptic calyx lobes (vs. triangular to semiorbicular), and white petals (vs. purplish-red).

#### Description.

Shrubs, 40–65 cm tall. Stems erect and branched, terete, sparsely puberulous with spreading, minute uniseriate hairs when young, often glabrescent when mature. Leaves opposite, equal or unequal; petiole 2.1–9 cm long, sparsely puberulous when young; blade ovate-cordate to ovate, 3.2–12 × 1.5–8.8 cm, thin papery, adaxial surface green and nitid, sometimes with white, orbicular patches when young, sparsely puberulous, glabrescent when mature, abaxial surface pale green, puberulous on veins, secondary veins 2 or 3 on each side of midvein, base cordate to subrounded, entire, inconspicuously and sparsely ciliate, apex acuminate. Inflorescence a terminal cyme, 1–8-flowered, peduncle 0.5–2.5 cm long, sparsely puberulous. Flowers bisexual, radial but androecium slightly bilateral, 4-merous, pedicels, hypanthium and calyx lobes puberulous; pedicles 0.5–1.7 cm long; hypanthium white to purplish-red, funnel-shaped, ca. 6–7 × 4–5 mm; calyx lobes 4, ovate-elliptic or elliptic, 5.5–7 × 3–4 mm, adaxially with a thick basal protuberance; petals 4, white, orbicular, 0.5–1.0 cm long, margin undulate and ciliate with glandular hairs, apex oblique and retuse; stamens 8 in two whorls, isomorphic, equal in length, filaments 6–7 mm long, bent with the anthers to one side of the flower, anthers lanceolate, 7–8 mm long, purplish-red, connective forming a 1.5 mm long, yellow dorsal spur and 2 yellow ventral lobes; ovary half inferior, locules 4, apex of ovary with membranous crown, crown margin ciliate with glandular hairs; style 0.7–1.5 cm long, basally sparsely puberulous. Capsule 7–9 × 6–7 mm, funnel-shaped; placentation axial, placentas non-thready; seeds numerous, ca. 1 mm long, cuneate.

#### Phenology.

Flowering June, fruiting late June to August.

#### Etymology.

The specific epithet is based on the nitid leaves.

#### Distribution.

*Bredianitida* is currently known from Hekou County, Yunnan Province, China (Fig. 2), occurring in moist forests on limestone slopes at 800–900 m at the Sino-Vietnamese border. Discovery of additional populations on the Vietnamese side is expected, as there are many lush limestone hills in the area.

### ﻿Key to karst species of *Bredia*

**Table:** 

1	Interveinal areas prominently bullate, each bulla with an apical seta	***B.bullata***
–	Interveinal areas flat	**2**
2	Petal margin entire; stamens ≤ 3 mm long	***B.reniformis***
–	Petal margin undulate; stamens > 5 mm long	**3**
3	Stem prostrate at least basally	**4**
–	Stem erect	**5**
4	Blade broadly ovate-cordate to cordate-orbicular, membranous and fragile, densely pubescent adaxially; petals white	***B.enchengensis***
–	Blade elliptic, oblong-elliptic, ovate to oblong-ovate or ovate-elliptic, papery, sparsely puberulous and strigose adaxially; petals pink	***B.longearistata*** / ***B.latisepala***
5	Stem broadly sulcate	**B.longiradiosavar.pulchella**
–	Stem not sulcate	**6**
6	Hypanthium setose, hair multiseriate and basally inflated	**B.longiradiosavar.longiradiosa**
–	Hypanthium puberulous, hairs uniseriate, not inflated basally	**7**
7	Stem and leaves densely pubescent; calyx lobes triangular to semiorbicular; petals purplish-red	***B.malipoensis***
–	Stem and leaves glabrescent when mature; calyx lobes ovate-elliptic or elliptic; petals white	***B.nitida***

## Supplementary Material

XML Treatment for Bredia bullata

XML Treatment for Bredia enchengensis

XML Treatment for Bredia nitida

## Supplementary materials

Supplementary material 1 Table S1 Data type Table Explanation note Source of materials studied and GenBank accession numbers for nrITS, *Dbr1*, *SOS4a*, and *trnV*–*trnM*. https://binary.pensoft.net/file/681722This dataset is made available under the Open Database License (http://opendatacommons.org/licenses/odbl/1.0/). The Open Database License (ODbL) is a license agreement intended to allow users to freely share, modify, and use this Dataset while maintaining this same freedom for others, provided that the original source and author(s) are credited.Jin-Hong Dai, Shi-Yue Nong, Xi-Bin Guo, Truong Van Do, Yan Liu, Ren-Chao Zhou, Ying Liu

Supplementary material 2 Table S2 Data type Table Explanation note Summary statistics of the optimal partition scheme and best-fitting model for each partition in phylogenetic analyses. https://binary.pensoft.net/file/681723This dataset is made available under the Open Database License (http://opendatacommons.org/licenses/odbl/1.0/). The Open Database License (ODbL) is a license agreement intended to allow users to freely share, modify, and use this Dataset while maintaining this same freedom for others, provided that the original source and author(s) are credited.Jin-Hong Dai, Shi-Yue Nong, Xi-Bin Guo, Truong Van Do, Yan Liu, Ren-Chao Zhou, Ying Liu
